# The systemic iron-regulatory proteins hepcidin and ferroportin are reduced in the brain in Alzheimer’s disease

**DOI:** 10.1186/2051-5960-1-55

**Published:** 2013-09-03

**Authors:** Animesh Alexander Raha, Radhika Anand Vaishnav, Robert Paul Friedland, Adrian Bomford, Ruma Raha-Chowdhury

**Affiliations:** 1 John Van Geest Centre for Brain Repair, Department of Clinical Neuroscience, University of Cambridge, Cambridge CB2 0PY, UK; 2 Department of Neurology, University of Louisville School of Medicine, Louisville, KY, USA; 3 Institute of Liver Studies, King’s College Hospital, London, UK

**Keywords:** Alzheimer’s disease, Amyloid, Brain iron homeostasis, Ferritin, Neurodegeneration, Vascular endothelium damage

## Abstract

**Background:**

The pathological features of the common neurodegenerative conditions, Alzheimer’s disease (AD), Parkinson’s disease and multiple sclerosis are all known to be associated with iron dysregulation in regions of the brain where the specific pathology is most highly expressed. Iron accumulates in cortical plaques and neurofibrillary tangles in AD where it participates in redox cycling and causes oxidative damage to neurons. To understand these abnormalities in the distribution of iron the expression of proteins that maintain systemic iron balance was investigated in human AD brains and in the APP-transgenic (APP-tg) mouse.

**Results:**

Protein levels of hepcidin, the iron-homeostatic peptide, and ferroportin, the iron exporter, were significantly reduced in hippocampal lysates from AD brains. By histochemistry, hepcidin and ferroportin were widely distributed in the normal human brain and co-localised in neurons and astrocytes suggesting a role in regulating iron release. In AD brains, hepcidin expression was reduced and restricted to the neuropil, blood vessels and damaged neurons. In the APP-tg mouse immunoreactivity for ferritin light-chain, the iron storage isoform, was initially distributed throughout the brain and as the disease progressed accumulated in the core of amyloid plaques. In human and mouse tissues, extensive AD pathology with amyloid plaques and severe vascular damage with loss of pericytes and endothelial disruption was seen. In AD brains, hepcidin and ferroportin were associated with haem-positive granular deposits in the region of damaged blood vessels.

**Conclusion:**

Our results suggest that the reduction in ferroportin levels are likely associated with cerebral ischaemia, inflammation, the loss of neurons due to the well-characterised protein misfolding, senile plaque formation and possibly the ageing process itself. The reasons for the reduction in hepcidin levels are less clear but future investigation could examine circulating levels of the peptide in AD and a possible reduction in the passage of hepcidin across damaged vascular endothelium. Imbalance in the levels and distribution of ferritin light-chain further indicate a failure to utilize and release iron by damaged and degenerating neurons.

## Background

Alzheimer’s disease (AD) is characterized by cerebrovascular and neuronal dysfunction leading to a progressive decline in cognitive functions and the development of dementia [1-3]. Pathological hallmarks of AD include neurofibrillary tangles consisting of hyper-phosphorylated microtubule-associated protein tau [4,5] and extracellular amyloid plaques derived from amyloid precursor protein (APP), a widely expressed trans-membrane metalloprotein essential for neuronal growth, survival, post-injury and repair [6]. The main component of plaques is amyloid β (Aβ) peptide, (38–43 amino acids) generated by sequential cleavage of APP by β- and γ-secretase [7-10]. Recently, it has been shown that oligomeric Aβ species (the smallest of which are dimers) isolated from AD brains are the most synaptotoxic forms found in amyloid plaques [11]. Another key protein involved in AD is apolipoprotein E (ApoE), a major genetic risk factor with 60-80% of affected individuals having at least one ApoE_4_ allele [12-15]. The majority of plasma ApoE is produced by hepatocytes, creating a hepatic pool that is important for lipid metabolism, while the second most common site of synthesis is the brain [16,17]. ApoE is an Aβ chaperone, promoting its transport across the blood brain barrier (BBB), a process that is known to be impaired in AD [18-20].

Iron homeostasis in the mammalian brain is important, yet poorly understood. Excess iron in the form of ferritin has been described in many neurodegenerative disorders including AD and furthermore there is an apparent link between an age-associated increase in iron stores in the brain and the increasing incidence of AD with advancing age [21-25]. Aβ and ferritin have been shown to co-localise in the vascular amyloid deposits of plaques in post-mortem AD brains [26,27] while iron accumulation in AD has been found to be a ready source of redox generated free radicals that promote neuronal cell death [28,29].

An increased understanding of how iron homeostasis is maintained at the whole body and cellular levels has followed from the identification of a number of iron related proteins [30-34]. Hepcidin is a regulatory hormone playing a key role in whole body iron homeostasis [30]. This liver-derived peptide regulates systemic iron homeostasis by controlling iron flux into the plasma from the duodenum as well as iron recycling macrophages through binding to its receptor, the iron exporter ferroportin [35-39]. Low serum hepcidin levels cause iron overload, as in haemochromatosis, while increased serum hepcidin expression plays an important role in the anaemia of inflammation by restricting intestinal iron absorption and macrophage iron release [40]. Hepcidin expression is modulated by systemic stimuli such as iron stores, hypoxia, oxidative stress and inflammation [31,41]. Ferroportin (FPN) is a transmembrane protein, (also known as SLC40A1, IREG1, MTP1) that exports iron from cells to plasma [42-44]. It is found on the surface of macrophages, Kupffer cells, hepatocytes, intestinal enterocytes and placental cells [32,45]. It is also localized in the brain in most cell types including neuronal perikarya, axons, dendrites and synaptic vesicles [46-49]. Recently, Duce and colleagues have reported that APP may bind to ferroportin to facilitate neuronal iron export and that disturbances in these processes may be implicated in AD brain pathology [50].

The aim of our study was to explore a possible role for these recently described proteins in the abnormalities of iron metabolism previously described in the brain in AD. Hepcidin and ferroportin proteins levels were assessed by Western blotting in AD brains as compared to age-matched controls. Cellular expression was investigated by immunohistochemistry of brain sections and comparisons were made with the distribution of AD markers Aβ and ApoE.

In conjunction with exploring the progressive abnormalities in iron homeostasis in AD, we also investigated age-associated changes in the expression of iron-handling proteins in the well-characterised APP transgenic (APP-tg) mouse (APP/PS1-tg2576) model [51-54]. We describe extensive blood vessel damage in AD brain and a reduction in hepcidin and ferroportin levels. In the APP-tg mouse model although the overall levels of ferritin in the brain were not increased there may have been a re-distribution of iron as an increase in ferritin immunoreactivity was found in the core of plaques.

## Methods

### Reagents and antibodies

Synthetic hepcidin and ferroportin peptides were purchased from Abcam, Cambridge. 4′,6-Diamidino-2′ phenylindole dihydrochloride (DAPI), 3,3′-diaminobenzidine (DAB) and methanol were from Sigma. Precast 4-12% NuPAGE BisTris minigel and prestained protein molecular weight markers were from Life technologies. Polyvinylidene difluoride (PVDF) membrane, Western blotting detection reagents (ECL Plus chemiluminescence reagents and Hyperfilm) were from GE Healthcare (UK). Vectastain ABC kit was from Vector laboratories (USA). Protease and phosphatase inhibitors were from Roche laboratories (Germany). BCA™ protein assay kit was from Thermo Scientific (USA).

### Sources of antibodies

The following primary antibodies were used: polyclonal anti-hepcidin, polyclonal SLC40A1, mouse monoclonal (MAB) anti-SLC40A1 (ferroportin), MAB anti-NG2, MAB anti-ApoE E6D7, Polyclonal anti-apoE, MAB anti-CD31, JC70A, Polyclonal anti-CD31, rabbit monoclonal anti-PDGF receptor β, from Abcam, (Cambridge, UK). The monoclonal anti-aβ antibody (6E10) (Covance, Princeton, USA), rabbit anti-aβ (1-40) (Millipore) and MAB anti-GFAP (Sigma). The following secondary antibodies were used: biotinylated goat anti-rabbit and biotinylated horse anti-mouse (both from Vector Laboratories, 1:250 for IHC); Alexa Fluor 568-labelled donkey anti-mouse, Alexa Fluor 488-labelled donkey anti-rabbit, and Alexa Fluor 568-labelled donkey anti-goat (all from Invitrogen, 1:1000 for IF). The dilutions of each antibody stock are given in Table 1.

**Table 1:** List of the primary antibodies used in this study

**Antibody**	**Species**	**Dilution**	**Supplier/cat. number**
Anti-β amyloid 1-16 amino acid (6E10)	Mouse (monoclonal)	1: 1000 for IHC	Covance cat number (SIG 39320)
		1:2000 for WB	
Anti-Hepcidin	Rabbit (polyclonal)	1: 200 for IHC	Abcam (Ab30760) (0.9 mg/ml)
		1:100 for WB	
Anti-Ferroportin (SLC40A1)	Mouse (monoclonal)	1: 1000 for IHC	Abcam (ab93438) (1 mg/ml)
Anti- Ferroportin (SLC40A1)	Rabbit (polyclonal)	1:200 for IHC	Abcam (ab85370) (1 mg/ml)
Anti-NG2	Mouse (monoclonal)	1:500 for IHC	Abcam (ab83508) (0.5 mg/ml)
Anti-NG2	Rabbit (polyclonal)	1:500 for IHC	Abcam (ab83178) (0.4 mg/ml)
Anti-GFAP	Mouse (monoclonal)	1:1000 for IHC	Sigma (G3893 Clone G-A-5)
Anti-GFAP	Rabbit (polyclonal)	1:200 for IHC	Abcam (ab48050) (1 mg/ml)
Anti-βIII-tubulin	Mouse (monoclonal)	1:1000 for IHC	Millipore (clone 2G10, neuronal | 05-559)
Anti β-actin	Mouse (monoclonal)	1:10000 for WB	Sigma (clone AC-74, A5316 )
Anti-APOE	Mouse (monoclonal)	1:1000 for WB	Abcam (ab1907) (1 mg/ml)
		1:500 for IHC	
Anti-APOE	Rabbit (polyclonal)	1:100 for WB	Abcam (ab85311) (0.1 mg/ml)
		1:100 for IHC	
Anti-Ferritin heavy chain	Rabbit (polyclonal)	1:200 for IHC	Abcam (65080) (1 mg/ml)
Anti-Ferritin light chain	Rabbit (polyclonal)	1:250 for IHC	Abcam (69090) (0.8 mg/ml)
Anti-Ferritin light-chain	Mouse (monoclonal)	1: 500 for IHC	Abcam (10060) (1 mg/ml)
		1:1000 for WB	
Anti-Myelin basic protein	Mouse (monoclonal)	1:500 for IHC	Abcam (ab62631) (1 mg/ml)
Anti-CD31 (JC/70A)	Mouse (monoclonal)	1:100 for IHC	Abcam (ab9498) (0.15 mg/ml)
Anti-CD31	Rabbit (monoclonal)	1: 1000 for IHC	Abcam (ab32457) (1 mg/ml)
Anti-PDGFβR1	Mouse (monoclonal)	1: 1000 for IHC	Abcam (ab32570) (1 mg/ml)

IHC, immunohistochemistry; WB, Western blotting.

### Human brain tissues

Human brain tissues from AD and age matched controls were provided by the UK Brain Bank and permission given by University of Cambridge, ethics committee for use of these tissues (Table 2). Autopsy tissue was examined from 6 adults (age 74.0 ± 8.1 years) with Alzheimer’s disease, meeting AD disease criteria (which include dementia, episodic memory loss). Comparison was made with six age-matched controls (age 82.0 ± 5.1 years) with no history of neurodegenerative diseases [55]. Due to limitations of tissue availability from some cases, it was not possible to include every case in all the analyses (below). Numbers included in the different analyses thus vary as indicated.

**Table 2:** Alzheimer’s disease cases and age matched control brain samples

**Case number**	**Category**	**Age**	**Gender**	**PM delay**	**Cause of death**
1	AD	83	Male	22.0	Bronchopneumonia
2	AD	80	Female	15.0	Cardiac failure
3	AD	74	Female	37.15	Gradual deterioration
4	AD	60	Male	12.9	Bronchopneumonia
5	AD	68	Male	17.5	AD, gradual decline
6	AD	81	Female	16.3	Aspiration pneumonia
7	Control	84	Female	31.45	Cancer, Heart failure
8	Control	81	Male	40.00	Chronic obstructive airway disease
9	Control	85	Male	43.35	Ca oesophagus
10	Control	73	Female	28.00	Ca bronchus
11	Control	83	Female	20.0	Bowel resection with complication
12	Control	88	Female	49.25	Chronic obstructive airway disease

AD = Alzheimer disease.

PM = post mortem.

Ca = carcinoma.

### Transgenic mice

APP-tg mice (Tg2576) over-expressing two human mutations (K670N and V717F) and one PS1 mutation (M146V), driven by Thy1 promoter were purchased from Jackson Laboratory, USA.

All animals were housed under standard conditions (12 h light-dark cycle, 20˚C ambient temperature) with free access to food and water. All procedures were performed under licence in accordance to the UK Animals (Scientific Procedures) Act 1986.

Neuropathological characterisation of these animals has previously been described in detail [51,52]. By 4-6 months of age, extensive amyloid plaque deposition is seen in hippocampus and cortical regions. Six mice from each age group (2, 4, 6, and 10 months-old) were used for immunohistochemistry and as described subsequently, six for protein quantitation by Western blotting. C57/bl mice were (n=6, age-matched to APP-tg mice) were used as control samples.

### Tissue preparation

In accordance with institutional guidelines for the humane treatment of animals, mice were terminally anaesthetised with carbon dioxide and culled. Unfixed tissues were carefully dissected from various brain regions from control and APP-tg mouse brains and snap frozen in dry ice until analysed by Western blotting. For histochemical analyses, animals were anesthetised with pento-barbitone and flash-perfused transcardially with 0.9% saline followed with 4% (v/v) paraformaldehyde (PFA) in 0.1 M phosphate buffer (pH 7.4). Brains were sectioned by microtome as described previously [56]. Free-floating sections were prepared (25 μm coronal sections in 0.1 M PBS) through the entire olfactory bulb, hippocampus, cortex, mid brain and cerebellum. Sections were then stained by immunohistochemistry as described below.

### SDS-PAGE and Western blotting

Levels of hepcidin, ferroportin, ApoE and aβ-42 (6E10) proteins were examined by Western blotting. Protein lysates were prepared from hippocampus and SVZ of control, and AD brain (n = 6) as described previously [57]. 20 μg protein samples were separated using a 12-20% gradient SDS-PAGE gel and transferred to polyvinylidene difluoride (PVDF, pore sizes 0.45 μm or 0.2 μm for hepcidin) membrane (Bio-Rad). The membrane was incubated with the appropriate primary antibody in blocking buffer (5% non-fat milk in 1 × tris buffer saline (TBS) for 24 h at 4˚C. The membrane was then washed three times with 1 × TBS plus 0.1% Tween 20 (1× TBST) and incubated for 1 hour at room temperature with HRP-conjugated secondary antibodies (anti mouse IgG, 1:3000, DAKO) or anti-rabbit IgG (1:3000; DAKO) antibodies). Finally, membranes were incubated with ECL Plus chemiluminescence reagents and were exposed to an X-ray film (Pierce). Levels of proteins were estimated by densitometry analysis using the Gel Analyzer module in the Image J program (NIH). Anti-actin immunoblot was used to normalise protein loading. Similarly protein lysates were prepared from cortex, hippocampus and SVZ of control and APP-Tg mouse brains (n = 6) and followed the same methodology.

### Immunohistochemistry

PFA fixed tissues were first quenched with 5% hydrogen peroxide and 20% methanol in 0.01M PBS for 30 min at room temperature followed by three rinses for 10 min in 0.01M phosphate buffer saline (PBS). Non-specific binding sites were blocked using blocking buffer (0.1 M PBS, 0.3% Triton-X100, and 10% normal goat serum for polyclonal antibodies or 10% normal horse serum for monoclonal antibodies) for 1 h at room temperature. Tissue sections were incubated overnight with the primary antibody diluted in blocking buffer (Table 1). Binding of the primary antibody was detected using a biotinylated secondary antibody followed by an avidin-biotin complex conjugated to peroxidase (Elite standard kit SK6100, Vector Laboratories) and DAB substrate (ABC substrate SK-4200, Vector Laboratories).

### Immunofluorescence

**Figure 1:**
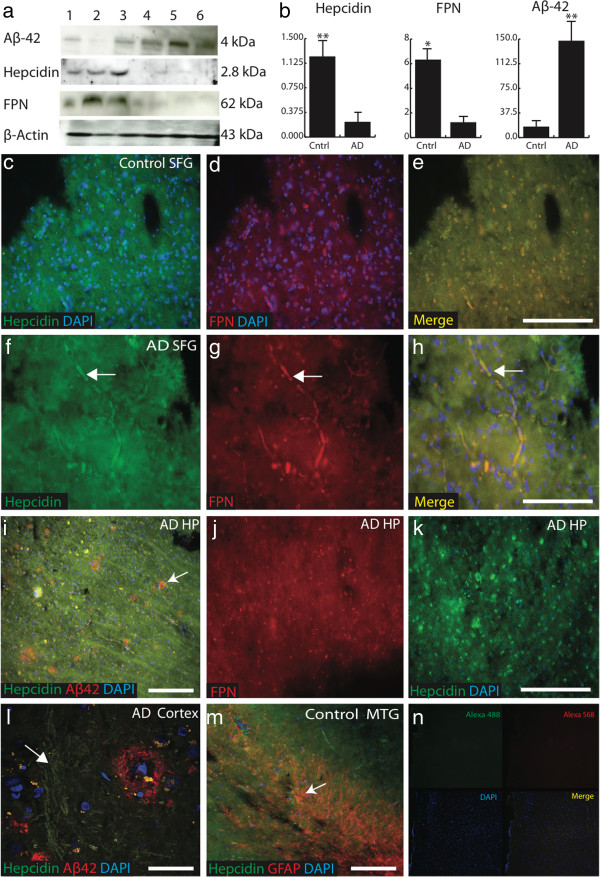
**Hepcidin and ferroportin protein levels decreased in AD brain. a.** Western blot analysis of the brain from frontal cortex demonstrated that a single 4 kDa band was clearly visible within all AD brain homogenates (lanes 4-6), in addition to a small proportion of control cortical tissue (lane 1-3). Ferroportin and hepcidin antibodies detected a single 62 kDa and 2.8 kDa band respectively in controls (lane 1-3), with less expression in AD. β-Actin loading control was used to normalize data. **b**. Densitometric analysis of the blots showed that there were significant differences between AD and control brains for all three peptides assessed. Aβ42 levels were higher in the diseased brain compared to controls (p<0.005) while hepcidin and ferroportin proteins were both significantly decreased (p<0.005 and p<0.01 respectively). **c**-**n**: Immunofluorescence in AD brains were assessed compared to healthy controls. Double staining was performed with polyclonal hepcidin and monoclonal ferroportin antibodies and counterstained with DAPI for nuclei. In the control brain superior frontal cortex hepcidin **(c)** and ferroportin **(d)** exhibited diffuse staining throughout the tissue. Both proteins co-localised in astrocytes **(e)**. In AD brain, both proteins were visible along the parenchymal surface of blood vessels **(f**-**g)**. Arrow indicates dual-labelling for hepcidin and ferroportin in small blood vessels **(f**-**h)**. In the hippocampus of AD brains, hepcidin was visible in neurons with limited co-localisation with Aβ **(**white arrow, **i**-**l)**. Ferroportin expression appeared to be reduced in AD brains **(j)**. However, hepcidin was visible in damaged neurons **(k)**. In control brain sections from mid temporal gyrus (MTG), hepcidin protein co-localised with GFAP **(m)**. To evaluate specificity of primary antibodies, a mouse brain section was incubated with secondary antibodies and DAPI, with the omission of the primary antibody. There was no visible non-specific binding **(n)**. *Scale bar* panels **c**-**e** and **i**-**k** &**m** 200 μm, **f**-**h** 100 μm, **l** = 25 μm.

Samples were blocked using blocking buffer (0.1 M PBS, 0.3% Triton X100, 10% normal donkey serum) for 1 hour at room temperature and incubated overnight with the primary antibody diluted in blocking buffer (Table 1). Alexa Fluor-conjugated secondary antibodies were used for detection, and samples were counterstained with 4′6-diamidino-2-phenylindole (DAPI, Sigma). Samples were then mounted on glass slides with coverslips using FluorSave (Calbiochem). All sections were processed simultaneously under the same conditions and the experiments are performed twice to assess reproducibility. To confirm the specificity of primary antibodies, control experiment was performed with sections incubated overnight in the absence of the primary antibody or with the primary antibody, that was pre-incubated with a blocking peptide (Figure 1n).

### Microscopy

Bright field images were taken and quantified using Lucia imaging software and a Leica FW 4000 upright microscope equipped with SPOT digital camera. Fluorescence images were obtained using a Leica DM6000 wide field fluorescence microscope equipped with a Leica FX350 camera and 20× and 40× objectives. Images were taken through several z-sections and deconvolved using Leica software. A Leica TCS SP2 confocal laser-scanning microscope equipped with 40× and 63× objectives was used to acquire high-resolution images.

### Image and statistics analysis

All experiments were performed in triplicate. Western blot and immunofluorescence images were quantified using ImageJ software (NIH). For Western blots, the gel analyser module was used. Selected bands were quantified based on their relative intensities, adjusted for background with fold-change in intensity subjected to statistical analysis as described below. Immunofluorescence was quantified using methods previously described [58]. Values in the figures are expressed as mean ± SEM. To determine the statistical significance, values were analysed by Student’s t-test when comparing difference between case (AD or APP-Tg brain) and control. A probability value of *p*<0.05 was considered to be statistically significant.

## Results

### Decreased hepcidin and ferroportin protein levels in AD brain

Analysis by Western blotting showed that Aβ42 levels were increased in diseased brain as compared to controls (P<0.005) while hepcidin and ferroportin proteins were both significantly decreased (P<0.005 and P<0.01 respectively, Figure 1a-b).

**Figure 2:**
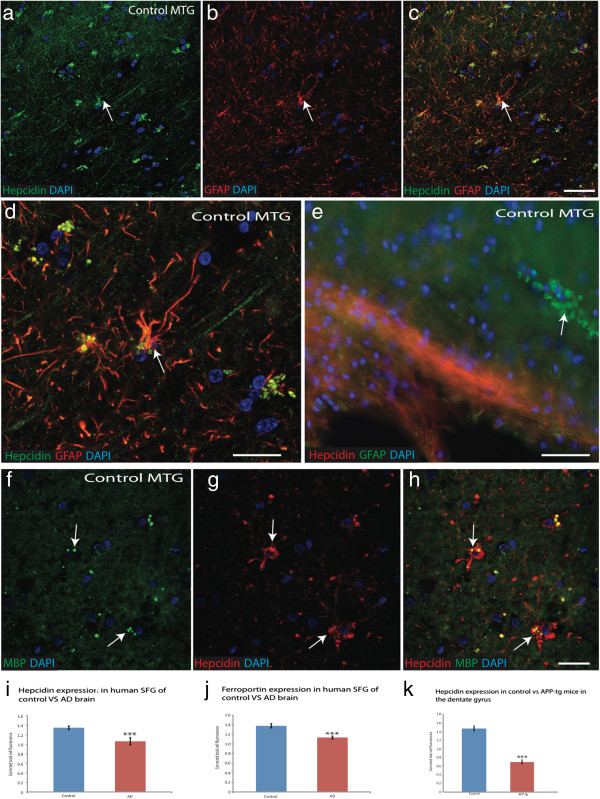
**Hepcidin protein present in glial cells.** In control brain sections from mid temporal gyrus (MTG) hepcidin expression was in astrocytes and co-localised with GFAP **(**white arrow, **a**-**d)**. Hepcidin was visible in red blood cells close to blood vessels **(e)**. Similarly in MTG in white matter tract hepcidin co-localised with myelin basic protein (MBP), suggesting that hepcidin protein is expressed in oligodendrocytes as well as in astrocytes **(f**-**h)**. *Scale bar* panels **a**-**c** and **e** 30 μm, d 15 μm, f-h 20 μm. **i**. Hepcidin protein levels in the human superior frontal gyrus (SFG) of AD brains compared to controls. Densitometric measurements of corrected total cell fluorescence adjusted against background (n=20, p<0.0001). **j**. Ferroportin protein level in human SFG of AD brain compared to controls (n=20, p<0.0001) **k**. Hepcidin protein level in the dentate gyrus of APP-tg mice vs control (n= 20, p<0.0001). ***denotes p<0.0001.

Brain sections from hippocampus, entorhinal cortex, and superior frontal gyrus were then analysed by immunofluorescence using antibodies specific to Aβ42 (6E10), hepcidin and ferroportin. In superior frontal gyrus and mid-temporal gyrus from control brains abundant hepcidin and ferroportin were present in neurons (Figure 1c-d) wherein the two proteins showed co-localization, while hepcidin predominated in astrocytes (Figure 1e). In AD brains extensive neuronal degeneration was observed and hepcidin and ferroportin staining was seen in the neuropil and in damaged blood vessels (Figure 1f-h). In sections of hippocampus from AD brains that were stained for hepcidin and ferroportin together with Aβ42, well established senile plaques were observed and hepcidin staining was also seen in association with fibrillary proteins (Figure 1i and l). Although there was marked heterogeneity of staining between different brain regions as well as between individual cases, overall, ferroportin protein was significantly reduced in AD brains as compared to control (Figure 1j) as evaluated by densitometric analysis (IMAGE J, Figure 2i-j, p<0.005). Hepcidin expression in other glial cells was evaluated in sections from the mid temporal gyrus, showing some co-localisation with the mature astrocytic marker GFAP in astrocytes close to blood vessels (Figure 1m). Glial cell types were then identified using confocal microscopy (Figure 2a-d, arrow) and in addition, hepcidin was present in white matter tracts in this region, where it co-localised with the mature oligodendrocytic marker MBP (Figure 2f-h). Hepcidin protein was also visible in red blood cells (Figure 2e, arrow).

### Hepcidin expression in neuritic processes and amyloid plaques

**Figure 3:**
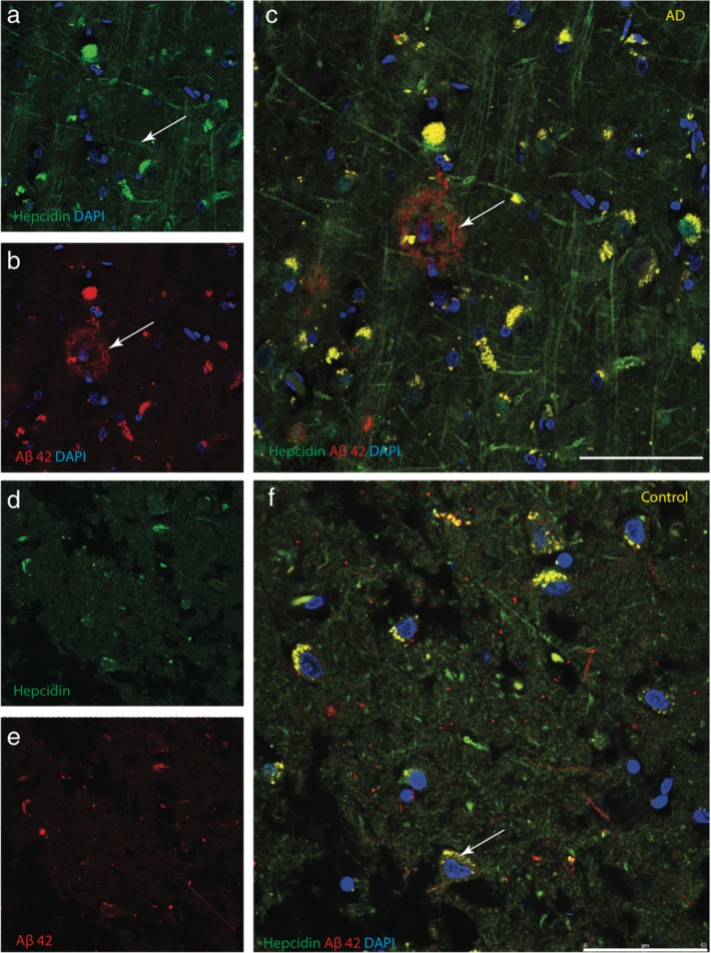
**Hepcidin expression in neuritic processes and amyloid plaques.** Confocal microscopic analysis of tissues from AD cases were immunostained for Aβ (6E10) and hepcidin antibody and counterstained with DAPI for nuclei (Blue). In AD brains, accumulation of hepcidin protein was visible in the intraneuronal compartment and in the abnormal fibrllary neuritic processes **(a)** and Aβ42 staining revealed characteristic plaques **(b)** in the hippocampus. In AD brains, the neuritic plaques with a wagon-wheel morphology and a DAPI positive nuclei **(c)**. Diffuse Aβ rich plaques occasionally presented with areas of hepcidin accumulations **(c)**. In the hippocampus of normal brains, hepcidin was present in pyramidal neurons of CA1, in the endosomal/lysosomal compartment **(d)**, minimal Aβ was visible in cell bodies **(e)** yet showed some co-localisation with hepcidin in perinuclear locations **(f)**. *Scale bar* a-f 25 μm.

For more precise co-localization of hepcidin with specific cellular markers as well as amyloid plaques, we examined brain sections by confocal microscopy. As shown in Figure 3, although uneven in its distribution in the hippocampus hepcidin appeared to be expressed in abnormal neuritic processes (Figure 3a). Scattered surviving neurons within the plaques also demonstrated hepcidin staining. Aβ42 staining revealed characteristic neuritic plaques with a wagon-wheel morphology having three components (i) an outer halo, (ii) an inner core and (iii) DAPI positive nuclei (Figure 3b-c). In contrast, in the hippocampus (CA1 region) of normal brain, hepcidin was present in pyramidal neurons, showing co-localisation with Aβ42 in an endosomal/lysosomal compartment (Figure 3d-f).

### Hepcidin and ferroportin protein expression in control mouse brain

**Figure 4:**
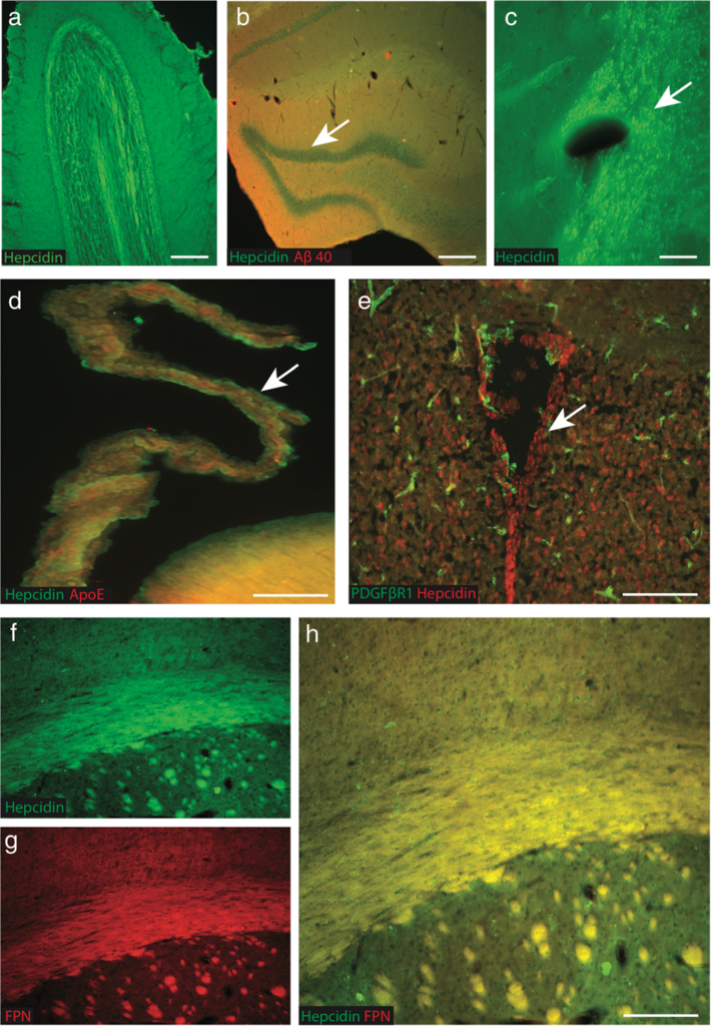
**Hepcidin and ferroportin protein expression in control mouse brain.** In control mouse brains, hepcidin protein was found in the molecular layer of olfactory bulb **(a)**, in the granule cells of dentate gyrus **(b)** and white matter tract of striatum **(c)**. Hepcidin and ApoE present in the epithelial cells of choroid plexus **(d)** and ependymal cells of the ventricular surface of lateral ventricle **(e)**. The immunoreactivity of oligodendrocytes was strong in white matter tracts, particularly in the corpus callosum (CC). Hepcidin **(f)** and FPN **(g)** expressed in the white matter tract of CC and both proteins co-localised **(h)**. *Scale bar***a**, **b** &**e** 150 μm, **c** 20 μm, d 50 μm, **f**-**h** 25 μm.

As shown by immunohistochemistry hepcidin protein was present in the mouse brain, observed in the olfactory bulb, hippocampus, granule cells of the dentate gyrus, striatum, choroid plexus and vascular endothelium of the lateral ventricles (Figure 4a-e). Ferroportin was present in olfactory glomerular neurons and in pyramidal neurons of the cortex (data not shown). Hepcidin immunoreactivity was also present in the white matter tracts of the olfactory bulb (Figure 4a) and corpus callosum, where it co-localised with ferroportin (Figure 4f-h).

### Hepcidin and ferroportin expression in APP-tg mice

**Figure 5:**
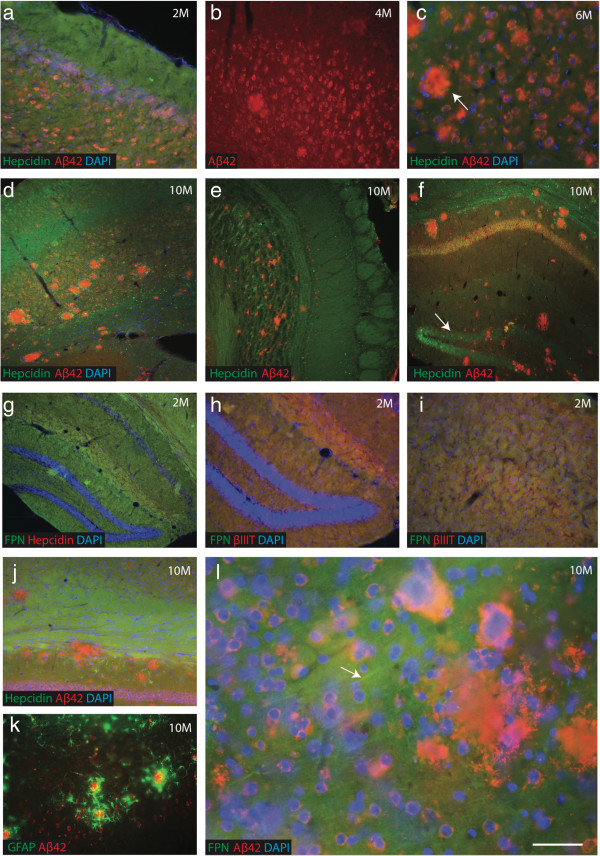
**Hepcidin and ferroportin expression in APP-tg mice.** Immunofluorescence staining of intraneuronal Aβ and intracellular hepcidin revealed an almost complete overlap in cortical neurons in two month old APP-tg mice **(a)**, as well as in cortical extracellular plaques in 4-6 month old APP-tg mice **(b**, **c** indicated with white arrow**)**. By 10 months, more severe pathology was evident with amyloid plaques increased in size and quantity and distributed throughout the cortex in close proximity to blood vessels **(d)**, olfactory bulb **(e)**, dentate gyrus, subiculum and the CA1 layer **(f)**. With disease progression, hepcidin levels were reduced in the dentate gyrus **(f**, white arrow**)**. At 2 months, FPN staining was seen in all cortical neurons, co-localising with the neuronal marker βIII tubulin **(g**-**i)**. Hepcidin was seen around the periphery of the maturing plaques **(j)** along with glial activation **(**identified by GFAP, **k)**, while with disease progression ferroportin levels were reduced with expression limited to fibrillary axons **(l**, white arrow**)**. *Scale bar***a**-**b** 150 μm, **c**-**g**, 100 μm, **j** 150 μm, **h**-**k** 50 μm, **l** 25 μm.

Prominent accumulation of intraneuronal Aβ was present in the frontal cortex, subiculum and CA1 of the hippocampus of APP-tg mice at 2 months age, with only very mild/no plaque pathology (Figure 5a). By 4 months, granular intraneuronal Aβ42 immunoreactivity was evident in pyramidal neurons (Figure 5b). In 6-month-old animals, plaque pathology increased in the frontal cortex (Figure 5c). By 10 months-old more severe pathology was evident with the amyloid plaques increased in size and quantity and distributed throughout the cortex in close proximity to blood vessels (Figure 5d-f).

Early on in the disease process (2-6 months), hepcidin was observed throughout the cortex, including the hippocampus, dentate gyrus, white matter tracts of the olfactory bulb and corpus callosum of APP-tg mice. With disease progression (10 months) hepcidin levels were reduced in the dentate gyrus (Figure 5f) as evaluated by densitometric analysis (IMAGE J, Figure 2k, p<0.0001). In sections from 10 months-old animals stained additionally for Aβ42, hepcidin was seen around the periphery of the maturing plaques (Figure 5d-f). In the early stages (2 months) extensive ferroportin staining was seen in the hippocampus (Figure 5g) and limited co-localisation with the neuronal marker βIIIT was observed in some cells (Figure 5h-i). Ferroportin levels were reduced as the disease progressed, with expression limited to axons (Figure 5l) while hepcidin expression was restricted to glial cells (astrocytes and oligodendrocytes) (Figure 5j-k).

**Figure 6:**
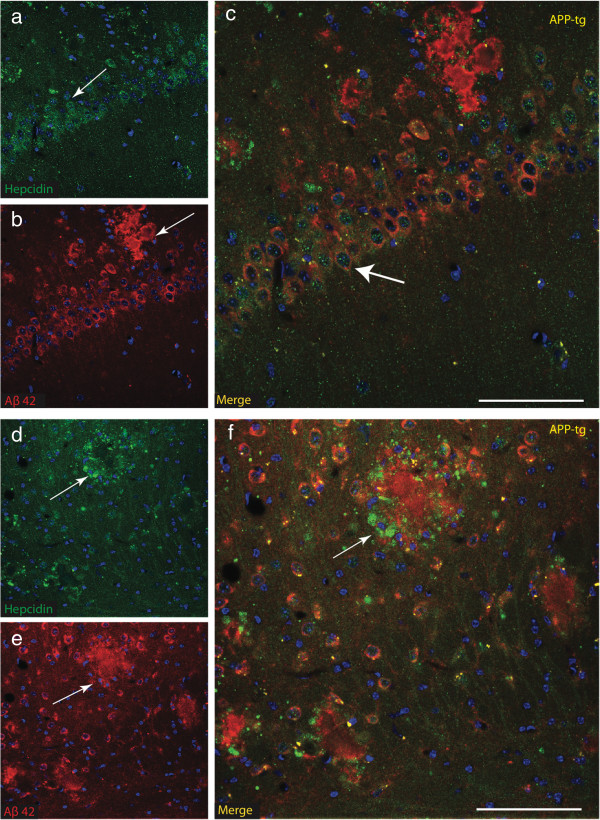
**Hepcidin protein in mature amyloid plaques in APP-tg mouse brain.** Confocal analysis of Aβ plaques and hepcidin co-localisation in brains from 10 month-old APP-tg mice. In the hippocampus (CA1), hepcidin granules were visible in the cytosol of selected neurons **(a)** and most of the neurons were positive for Aβ42 **(b**-**c)**. Individual deposits were analysed by confocal laser scanning microscopy and z-stacks were prepared. Hepcidin was present in surviving neurons in the DG, distributed around the periphery of amyloid plaques in a form of a “halo” indicating wagon-wheel plaques **(d**-**f)**. Arrowheads indicate dual-labeled plaques. *Scale bar* a-f 25 μm.

To further investigate the location of hepcidin protein in mature amyloid plaques (10 months) sections were stained with Aβ42 (6E10), hepcidin and DAPI, and examined by confocal microscopy. In the hippocampus (CA1), most of the neurons were positive for Aβ42, with hepcidin granules visible in the cytosol of selected neurons (Figure 6a-c). Although hepcidin was associated with fibrillary protein in AD brains (Figure 3a and c), no such proteins were seen in APP-tg mice (Figure 6a-c). At higher magnification, hepcidin was present in surviving neurons in the dentate gyrus, distributed around the periphery of amyloid plaques in the form of a “halo” (Figure 6d-f) similar to the distribution of BMP6, as described by others [59].

### Ferritin accumulation in the APP-tg mouse brain during disease progression

**Figure 7:**
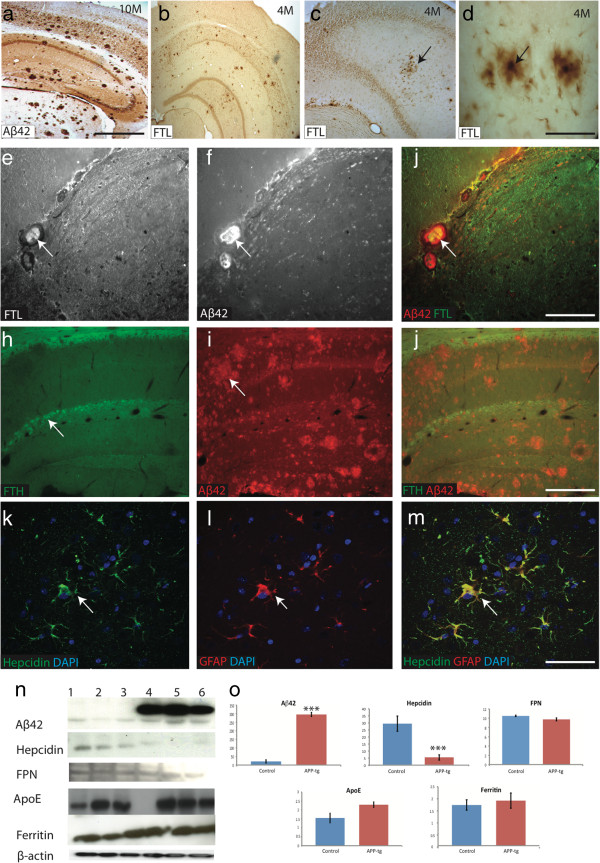
**Ferritin accumulation in the APP-tg mouse brain.** DAB immunostaining of 10 month-old APP-tg mice showed intraneuronal and extracellular accumulation of Aβ peptides present throughout the brain, including in the hippocampus, dentate gyrus and all six layers of cortex **(a)**. Another section from cortex stained with ferritin light chain (FTL) showed immunoreactivity throughout cortex, as well as in plaques **(b**-**c)** and centre core of the plaques **(d)**. Double-label IFC staining of Aβ42 and FTL, seen close to the blood vessels in the hippocampus and both proteins were detected throughout the brain **(e**-**g)**. Ferritin heavy chain (FTH) was visible in a selected population of neurons in the hippocampus but did not co-localise with Aβ42 **(h**-**j)**. Hepcidin was seen in GFAP positive astrocytes **(k**-**m)**. Protein levels were quantified by Western blotting from 6 month-old APP-tg mice and age matched controls (n = 6). Aβ42 levels were higher in APP-tg mice compared to control (p < 0.0002) while hepcidin levels were decreased (p<0.005). Ferroportin, ApoE and FTL levels, however, did not significantly change **(n**-**o)**. *Scale bar***a**-**b** 250 μm, **c** 100 μm, **d** 25 μm, **e**-**j** 75 μm, **k-m** 30 μm.

With increasing plaque formation in the frontal cortex (Figure 7a), ferritin light-chain (FTL) accumulated in the core of plaques, as seen in sections from a 10 month-old animal (Figure 7b-d). To further assess the distribution of ferritin, brain sections (6 months) were stained with Aβ42, FTL and ferritin heavy-chain (FTH) by immunofluorescence. FTL was widely distributed throughout the brain showing co-localisation with Aβ42 particularly in the vicinity of blood vessels and ventricles (Figure 7e-g). FTH expression was limited to hippocampal neurons but did not co-localise with Aβ42 (Figure 7h-j). During the early stages of plaque formation (4-6 months), GFAP-positive activated astrocytes that were seen surrounding neuritic plaques demonstrated co-localisation with hepcidin (Figure 7k-m).

Finally, protein steady state levels were measured and compared by Western blotting using brain tissue (cortex including hippocampus and dentate gyrus) from 6 month-old APP-tg mice and age-matched controls (n = 6). Aβ42 levels were higher in APP-tg mice compared to control (p<0.0002) while hepcidin levels were decreased (p<0.005). However significant changes were not seen in levels of ferroportin, ApoE and FTL (Figure 7n-o).

### Vascular endothelial damage in the APP-tg mouse

**Figure 8:**
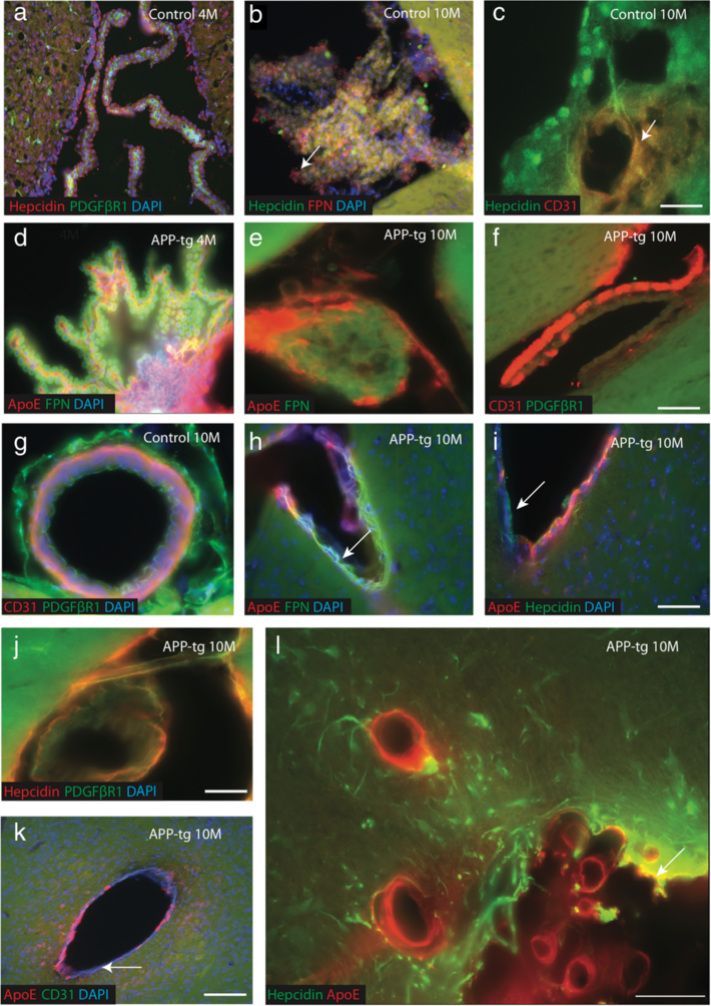
**Vascular endothelial damage in the APP-tg mouse.** Immunofluorescence staining of hepcidin and ferroportin with endothelial markers CD31 and PDGFβR1 was performed using brain sections from lateral ventricles, choroid plexus and blood vessels. In control mice, hepcidin staining was detected in ependymal cells of lateral ventricles at 4 months **(a)**, the choroid plexus at 10 months-old where hepcidin and FPN both co-localised in epithelial cells, with higher FPN expression on the ventricular side **(**white arrow, **b)**. Hepcidin was present in endothelial cells in blood vessels **(c)**. Hepcidin staining was visible in epithelial cells of choroid plexus where as ApoE was found in the blood vessel wall **(c)**. There was no vessel damage noticed in 4 months old APP-tg mouse choroid plexus stained with ApoE and FPN **(d)**, whereas by 10 months more vascular damage was apparent **(e)**. Similarly sections close to the lateral ventricle of APP-Tg mice were stained with ApoE and FPN **(h)**, ApoE and hepcidin **(i)** indicating extensive endothelial damage. Blood vessel integrity was assessed with CD31 and PDGFβR1 markers, with intact endothelium and pericytes were observed in controls **(g)** while badly damaged endothelia was seen in APP-tg mice at 10 months **(f)** and **(j)**. In another section from APP-tg thalamus stained with ApoE and CD31 showed a complete loss of endothelial layer at 10 months **(k)**. Hepcidin and ApoE co-localised in large blood vessels **(l)**. Scale bar **a**-**b**, 100 μm, **c** 25 μm, **d**-**f** and **h**-**k** 75 μm, **g** and **l** 50 μm.

ApoE and Aβ are known to be cleared from the central nervous system by efflux across the blood brain barrier and this process is impaired in AD as a consequence of vascular damage [18,55]. The expression of hepcidin and ferroportin was therefore investigated specifically in the vascular endothelium of the APP-tg mouse model. In control mouse brain, the vascular endothelium was characterised by strong hepcidin expression as shown previously (Figure 4e) and also by the presence of ferroportin that co-localised with hepcidin in the choroid plexus (Figure 8b) and with PDGFβR1 in the endothelium of lateral ventricles (Figure 8a). Co-localisation was also noted between CD31 and hepcidin in the endothelial lining of the blood vessels (Figure 8c). In 4-month-old APP-tg mice, vascular damage was not evident and co-localisation between ApoE and ferroportin was seen in the endothelium of the choroid plexus and lateral ventricles (Figure 8d).

By 10 months APP-tg mice showed widespread endothelium damage, loss of cellular integrity and, in some sections, loss of endothelium in the wall of ventricles (Figure 8e-i). Sections were also stained with CD31 (endothelium marker) and a pericyte marker (PDGFβR1) to confirm the appropriate staining pattern in controls (Figure 8f-k) and extensive endothelial disruption in the APP-tg mouse model (Figure 8e-k). Both hepcidin and ApoE were found in APP-tg mice close to the blood vessels (Figure 8l).

### Hepcidin co-staining with haem-rich deposit and red blood cells in AD brain

**Figure 9:**
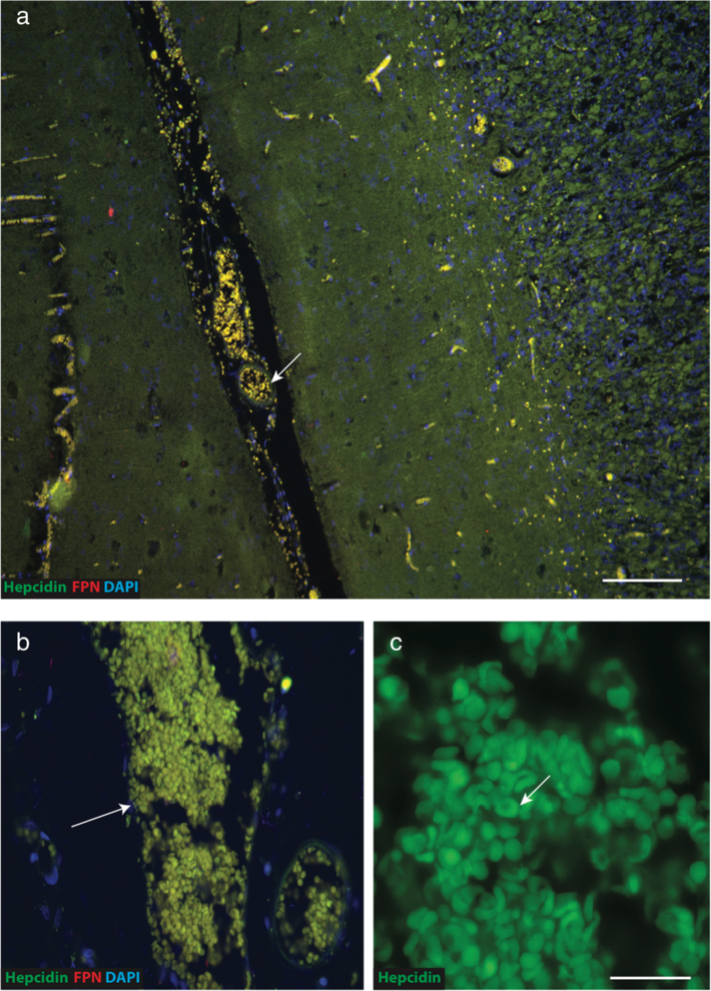
**Hepcidin visible in haem-rich deposit and red blood cells in AD brain.** AD (SFG) human brain sections double-labeled with hepcidin and ferroportin by IFC and counterstained with DAPI. Limited hepcidin and FPN protein expression was seen throughout brain parenchyma with some autofluorescent puncta due to haem rich deposits **(**HRD, **a)**. Higher magnification showed both proteins in HRD, visible in small arterioles **(b)**. A single labelled image showed RBCs with typical biconcave morphology were also positive for hepcidin **(c)**. Scale bar: **a** 200 μm, **b** 50 μm, **c** 15 μm.

Haem-rich deposits and red blood cells were scattered throughout the parenchyma in AD brain in accordance with previously reported observations [60]. We also noted a close relationship of haem-rich deposits to small blood vessels in all cases examined (Figure 9a). Red blood cells demonstrating typical biconcave morphology within large blood vessels co-localized with hepcidin, while a faint ferroportin signal was observed (Figure 9c, marked with arrow). Intravascular red blood cells appear yellow in the figure resulting from a combination of red haem colour and green signal from hepcidin (Figure 9a-b).

## Discussion

The pathological features of the common neurodegenerative conditions, Alzheimer’s disease, Parkinson’s disease and multiple sclerosis are all known to be associated with iron dysregulation in regions of the brain where the specific pathology is most highly expressed [21,25]. The diversity of the pathological processes involved make it unlikely that there is a primary abnormality of brain iron metabolism common to these diseases, although this is the case with a group of rare genetic disorders characterized by neurodegeneration with brain iron accumulation (NBIA), [61]. Even if the abnormalities in iron metabolism in common neurodegenerative disorders are secondary phenomena the finding that iron-related oxidative damage in Alzheimer’s disease is an early event in the disease process [29,62] suggests that the control of iron levels in the brain remains a worthwhile therapeutic target [63].

In the present study the expression of proteins that play a central role in maintaining systemic iron homeostasis, hepcidin and its receptor, ferroportin, was investigated in human AD brains and in the APP transgenic mouse model to further characterize abnormalities of iron metabolism. Hepcidin and ferroportin protein were found to be widely distributed in normal human and mouse brain but levels were decreased significantly in AD brains and in the later stages of the mouse model. The expression of ferroportin protein has been reported to be decreased by ischaemia [64,65] and inflammation in the rat cortex [66,67] and in primary cultures of rat brain cells [68,69]. The down-regulation of ferroportin by inflammatory stimuli in cells derived from the brain mirrors the findings in multiple cell types in systemic iron metabolism [70-72]. The primary pathology of AD, that of protein misfolding [5,10], is accompanied by other pathological processes, notably vascular damage with associated ischaemic changes [73,74] and inflammation [75,76] and we believe that the reduction in ferroportin expression found in the present study is likely to be a secondary phenomenon caused by these factors that clearly contribute to AD pathogenesis [77,78]. Ferroportin is also down-regulated when bound by hepcidin at the cell surface, an event that leads to the internalization and degradation of the iron carrier [35]. This was observed in rat brain when the expression of ferroportin was reduced following the intra-ventricular administration of hepcidin [79,80] or when hepcidin was added directly to primary cultures of neurons, astrocytes and microglia [69]. We found that hepcidin levels were reduced in human and mouse brains exhibiting severe AD pathology but early in the course of the disease, as shown in the mouse model, hepcidin levels did not differ significantly from controls and the interaction with ferroportin as seen in cortical neurons by immunohistochemical staining could contribute to the decline in levels of the iron carrier. Inflammation in AD [75] could be a further reason for the increase in hepcidin levels as in the systemic environment hepcidin synthesis by hepatocytes is transcriptionally regulated by IL-6 through the STAT-3 signalling pathway [81]. Interestingly in the dentate gyrus of the APP mouse and in AD brains we found that hepcidin was distributed around the periphery of amyloid plaques and in surviving neurons, in a similar distribution to that of IL-6 around plaques and in large cortical neurons reported previously in AD [82].

The finding that hepcidin and ferroportin were co-localised in cortical neurons in control brains is consistent with a role in for these protein in regulating neuronal iron release [35]. However, neither the constitutive loss of hepcidin through gene mutations in either human [83] or mouse models of haemochromatosis [84,85] or the targeted loss of ferroportin in the brain [45] appear to cause cerebral or cerebellar dysfunction and a role for hepcidin and ferroportin in the brain is currently undefined. Our finding that hepcidin protein was widely distributed in normal human and mouse brain is consistent with previous reports [64,86] and raises the question of the origin of this protein given that hepcidin mRNA was not consistently detected by *in situ* hybridization in normal mouse brain [86]. Hepcidin is a gene-encoded antimicrobial peptide structurally related to members of the defensin and protegrin families [87], most of which are cationic, a property that facilitates adsorption and insertion into anionic bacterial cell walls [87]. Cationic peptides also cross mammalian cell membranes [88] and the blood brain barrier [89-91] and it is possible that hepcidin may cross the vascular endothelium to enter the brain interstitium.

Direct evidence for iron mishandling in AD brain comes from the histochemical demonstration of non-haem iron deposits in senile plaques [21,92,93] and Aβ plaques in APP mice [94] and iron levels were also found to be increased in neurofibrillary tangles and plaques using laser microprobe mass analysis [95] and particle-induced X-ray emission [96]. It is not clear whether this represents increased deposition of iron and other transition metals [97] in the region of plaques or a more general increase as iron levels have not been found to be consistently increased in AD brains [98-100] compared to age-matched controls. Increased immunoreactivity for the iron storage protein ferritin, within and around plaques [101,102] is further evidence of a local increase in iron as this protein is regulated primarily at the translational level through the binding of iron regulatory proteins to ferritin H- and L-chain mRNAs [34]. Consistent with these findings in human AD brains, we found strong immunoreactivity for ferritin L-chain in maturing plaques in the later stages of the APP mouse while early on in the disease process this isoform was widely distributed throughout the brain in association with Aβ42 in the vicinity of blood vessels. There is recent evidence that ferritin L-chain may have a fundamental role in plaque pathology by binding to and stabilising PEN-2, a functional component of γ-secretase, the enzyme that cleaves APP to generate Aβ [103]. Building on this observation it has been suggested that increased levels of iron and, hence, ferritin L-chain may lead to increased production of Aβ [34,103]. The excess iron in plaques and associated increase in ferritin L-chain, the iron-storage isoform [34], is likely a secondary event resulting from a failure to utilise iron by dead and dying neurons. The suggestion that ferritin L-chain may lead to increased production of Aβ by increasing the activity of γ-secretase [103] would be consistent with a role for iron in promoting and maintaining plaque pathology. In agreement with earlier reports [25,104] expression of ferritin H-chain was restricted to pyramidal neurons of the hippocampus.

Recent studies into the cause of vascular dysfunction in neurodegenerative diseases such as AD [73] suggest that the mechanisms include breakdown of the blood-brain barrier as a result of loss of pericytes [2,73,105], hypo-perfusion leading to hypoxia and brain ischaemia [74] and endothelial dysfunction [77]. Furthermore, these abnormalities in vascular structure and function were recapitulated in the arcAβ mouse model of AD [106] while in the late stages of the APP-tg model used in the present study, loss of pericytes and extensive endothelial disruption was seen confirming the presence of severe vascular pathology. Loss of vascular integrity is also responsible for abnormal iron accumulation in addition to ferritin in AD brains in the form of haem-positive granular deposits. These have been demonstrated in aged brains in association with senile plaques and result from capillary bleeds or micro-haemorrhages [60,107]. In AD brains where extensive neuronal damage was present, although levels of hepcidin and ferroportin were reduced, both proteins were found in association with haem-positive granular deposits in the region of damaged blood vessels.

## Conclusions

Our results describe the progressive changes and dynamic interplay of iron homeostasis, vascular changes and neuronal degeneration in AD. In conclusion, as vascular damage with associated ischaemic change is widespread in AD brains it is likely that the reasons for a reduction in ferroportin levels are multifactorial but include cerebral ischaemia, inflammation, the loss of neurons due to the well characterised protein misfolding, senile plaque formation and possibly the ageing process itself. Progressive accumulation of ferritin light chain in the core of amyloid plaques of the APP tg mouse implies that there is an imbalance in iron utilization and release.

## Abbreviations

AD: Alzheimer’s disease; Aβ: Amyloid-β-peptide; ApoE: Apolipoprotein E; APP: Amyloid precursor protein; APP-tg: APP transgenic mouse; βIIIT: β Tubulin type III; BSA: Bovine serum albumin; CA: Cornu ammonis; CP: Choroid plexus; CVD: Cerebrovascular dementia; DG: Dentate gyrus; FTL: Ferritin light chain; FTH: Ferritin heavy chain; FPN: Ferroportin; IF: Immunofluorescence; IHC: Immunohistochemistry; NFT: Neurofibrillary tangles; OB: Olfactory bulb; PAGE: Polyacrylamide gel electrophoresis; PBS: Phosphate buffered saline; PD: Parkinson’s disease; SDS: Sodium dodecyl sulphate; SFG: Superior frontal gyrus; WB: Western blotting.

## Competing interests

The authors declared that they have no competing interests.

## Authors’ contributions

AAR performed all experiments, including characterization of APP-tg mouse model and confocal microscopy. RAV and RPF contributed to the hypothesis development and implications of vascular defect in AD brain and edited the manuscript. AB and RRC contributed to the hypothesis development, performed study design, critically evaluated the results and wrote the manuscript. All authors read and approved the final manuscript.
